# Phospholipase C Beta 2 as a Key Regulator of Tumor Progression and Epithelial-Mesenchymal Transition via PI3K/AKT Signaling in Renal Cell Carcinoma

**DOI:** 10.3390/biomedicines13020304

**Published:** 2025-01-26

**Authors:** Shijin Wang, Deqian Xie, Hongzhe Yue, Guandu Li, Bowen Jiang, Yaru Gao, Zunwen Zheng, Xu Zheng, Guangzhen Wu

**Affiliations:** 1 Department of Urology, The First Affiliated Hospital of Dalian Medical University, Dalian 116011, China; 2 Department of Emergency, The First Affiliated Hospital of Dalian Medical University, Dalian 116011, China; 3 Department of Nursing, The Second Affiliated Hospital of Dalian Medical University, Dalian 116011, China; 4 Department of Cell Biology, College of Basic Medical Science, Dalian Medical University, Dalian 116011, China

**Keywords:** phospholipase C Beta 2, epithelial-mesenchymal transition, signaling pathway crosstalk, renal cell carcinoma, multi-omics, predictive biomarkers

## Abstract

**Background:** Renal cell carcinoma (RCC) represents the most common form of invasive kidney cancer in adults. Among the components critical to cellular regulation is Phospholipase C Beta 2 (*PLCB2*), a member of the phospholipase C enzyme family. This enzyme plays a vital role in managing key cellular functions such as growth, differentiation, migration, and survival. Despite its significant importance, the specific expression patterns and molecular mechanisms of *PLCB2* in the progression of RCC are not well understood. **Methods:** This investigation employed a combination of bioinformatics analyses, scRNA-seq, functional assays, transcriptome sequencing, real-time quantitative PCR (RT-PCR), immunofluorescence, rescue experiments, and Western blotting to explore the regulatory function of *PLCB2* in driving the epithelial-mesenchymal transition (EMT) in RCC through the PI3K/AKT signaling pathway. **Results:** *PLCB2* expression is significantly elevated in RCC samples, and this increase is inversely correlated with patient prognosis. The knockdown of *PLCB2* in RCC cell lines leads to a marked reduction in cell proliferation, invasion, migration, and EMT. Transcriptome sequencing further revealed that *PLCB2* is significantly associated with the PI3K/AKT pathway. Notably, the PI3K activator 740Y-P was able to reverse the reductions in migration, invasion, and EMT caused by the *PLCB2* knockdown. **Conclusions:** Our findings underscore the pivotal role of *PLCB2* in regulating RCC invasion and metastasis by modulating the EMT via the PI3K/AKT signaling pathway. This highlights *PLCB2* not only as a key prognostic biomarker, but also as a promising therapeutic target in the treatment of advanced-stage RCC, offering new avenues for more effective interventions.

## 1. Introduction

RCC ranks as the ninth most common cancer worldwide, affecting about 400,000 people annually [1]. Despite the advancements in imaging techniques and novel treatments, the prognosis for many RCC patients remains poor. Surgical resection, targeted therapies, and immunotherapies have contributed to early detection and improved management. However, up to 30% of early-stage patients experience relapse. Additionally, nearly a quarter of cases progress to metastatic disease, significantly reducing survival rates [2,3]. These limitations highlight the urgent need for novel therapeutic strategies that address the mechanisms driving the RCC progression. Specifically, a deeper understanding of the molecular pathways involved in RCC pathogenesis is critical to improving outcomes, particularly for advanced-stage patients.

Among the myriads of molecular players in cancer biology, phospholipase C Beta 2 (*PLCB2*), a member of the phospholipase C family, located on chromosome 4, has emerged as a potential candidate in cancer progression. *PLCB2* plays pivotal roles in cell proliferation, differentiation, and metabolism through the activation of protein kinase C (PKC). Additionally, it is activated by G-protein-coupled receptors and participates in signal transduction via type 2 taste receptors [4]. Research indicates that nuclear factor kappa B (NF-κB) regulates the transcription of the *PLCB2*, and its protein product is also a key regulator in platelet function [5]. Recent studies show that *PLCB2* plays significant roles in various tumor types. For instance, in melanomas, *PLCB2* induces tumor cell apoptosis via RAS/RAF/MAPK pathway activation [6]. It also regulates *VEGF*-induced vascular permeability by controlling intracellular calcium levels [7]. In breast cancer, *PLCB2* has been shown to promote progression through the G2/M phase of the cell cycle and to regulate mitosis [8]. Despite these findings, the precise biological roles and molecular mechanisms of *PLCB2* in RCC remain poorly understood. This lack of knowledge presents a critical gap that limits advancements in RCC treatment, particularly for the advanced or metastatic disease.

One key mechanism, connecting signaling pathways to tumor progression, is EMT, a process that promotes metastasis and poor clinical outcomes [9,10]. During EMT, cancer cells often lose epithelial markers such as E-cadherin, which are vital for cell–cell adhesion, and gain mesenchymal markers, like N-cadherin and Vimentin, which boost their migratory and invasive abilities [11,12,13]. Understanding how specific molecules, including *PLCB2*, influence EMT within the RCC microenvironment is crucial for revealing new therapeutic vulnerabilities.

Cancer signaling does not occur in isolation; it is governed by a complex network of interconnected pathways, including Wnt and PI3K/AKT. These pathways are central to cellular homeostasis, and their dysregulation can drive tumor initiation, growth, and resistance to therapy [14,15]. Wnt signaling, in particular, regulates proliferation, differentiation, and apoptosis [16,17]. Aberrant activation of Wnt3a and other ligands can lead to malignant transformations and poor prognoses in various cancers [18,19,20]. Similarly, the PI3K/AKT pathway underpins cell survival and therapy resistance, inhibiting apoptosis and promoting tumor aggressiveness [21,22,23]. By examining the interactions of *PLCB2* within these pathways and their cross-communication, this study seeks to reveal critical molecular drivers of RCC progression, offering potential therapeutic insights.

In this study, we aimed to elucidate the role of *PLCB2* in RCC using a comprehensive approach combining bioinformatics and molecular functional studies. Through a pan-cancer analysis of 117 Wnt-signaling pathway genes, we investigated their copy number variations (CNVs), single nucleotide variants (SNVs), and expression patterns. Focusing specifically on kidney renal clear cell carcinoma (KIRC), we identified correlations between the expression of Wnt pathway genes, immune infiltration, and drug sensitivity. Our findings revealed that *PLCB2* is notably upregulated in RCC and correlates with poor patient prognosis. Further high-throughput RNA sequencing and molecular assays demonstrated that *PLCB2* acts as a key mediator connecting the Wnt and PI3K/AKT pathways to regulate the EMT, critically influencing RCC cell proliferation, migration, and invasion. These results underscore the potential of *PLCB2* as a therapeutic target and provide insights into its role in RCC progression.

## 2. Materials and Methods

### 2.1. Data Acquisition

We identified 117 Wnt signaling pathway *genes* from the M39669 gene set available on the gene set enrichment analysis (GSEA). The genomic data for 32 cancer types, including gene expression profiles and mutation information, were sourced from The Cancer Genome Atlas (TCGA) database [24,25]. Perl programming was used for processing gene expression data, and TBtools(version2.153) software for visualization. We utilized R packages such as ‘Seurat(version 5.1.0)’ and ‘SingleR’(version 2.4.1) to analyze the renal cancer single-cell dataset GSE152938. To ensure the retention of high-quality single-cell RNA sequencing (scRNA-seq) data for the subsequent analysis, the following filters were applied: cells with less than 300 feature counts, cells with more than 25% of mitochondrial genes and less than 3% of ribosomal genes, and doubled cells were removed. We used the “harmony” package (version 1.2.3) to eliminate the batch effects and set the resolution to 0.5 for cell clustering. Cell annotation was performed using SingleR and CellMarker (http://xteam.xbio.top/, accessed on 13 October 2024).

### 2.2. Clustering Based on the Expression of the Wnt Gene

We developed a Wnt scoring model to assess the Wnt pathway gene expression in kidney cancer samples. Tumor samples were classified into three clusters: active (Cluster 1), normal (Cluster 2), and inactive (Cluster 3) Wnt expression groups, based on the comparisons with normal samples. We utilized violin plots to display gene enrichment scores for each cluster. The ‘ggplot’ package(v3.3.6) in Rstudio(version 4.3.2) was employed for clustering visualization, and survival curves for the clusters were plotted using the ‘Survival’ package (version 3.3.1), with a statistical significance threshold set at *p* < 0.05.

### 2.3. Researching Drug Sensitivity and Targeting Pathways in KIRC

To predict the efficacy of pazopanib, sunitinib, and nilotinib in treating RCC, we utilized the Genomics of Drug Sensitivity in Cancer (GDSC) database [26], a comprehensive resource of genomic data and drug response information. Drug efficacy predictions were made using the ‘pRRhetic’ R package (version 0.5), which provided IC50 estimations for these agents [27,28]. Lower IC50 values suggest a higher drug potency at given concentrations, potentially indicating effectiveness in inhibiting KIRC cell growth. The reliability of our predictions was confirmed through 10-fold cross-validation, with a *p*-value of less than 0.05 considered statistically significant. Further exploring targeted therapies, we utilized the connectivity map (CMap) platform [29], where we analyzed drug targets and their mechanisms of action.

### 2.4. Analyzing the Correlations Between Oncogenes, SIRTs, HDACs, and Wnt Pathway Genes in KIRC

To elucidate the correlations between classical oncogenes, sirtuins (SIRTs), histone deacetylases (HDACs), and Wnt pathway genes in RCC, we employed a heatmap analysis. Our findings were considered statistically significant with *p*-values less than 0.05.

### 2.5. Infiltration of Immune Cells and Immunotherapy

We used a single-sample gene set enrichment analysis (ssGSEA) and TCGA database expression data to investigate the relationship between Wnt pathway genes and immune cell infiltration in KIRC. Correlation heatmaps, created using the ‘ggplot2’ R packages, displayed interactions among 29 immune cell types and regulators from both innate and adaptive immune systems. Key immunomodulators—such as mast cells, parainflammation responses, regulatory T cells (Tregs), and type II interferon (IFN) responses—were analyzed for their correlation with Wnt gene scores, visualized in scatter plots produced with the ‘GGSCATterStats’ R package (version 0.12.1).

We further investigated the interactions between immunotherapy targets, such as PD-1 and CTLA-4, and the Wnt pathway. These interactions were visualized in a correlation grid plot with a color gradient showing correlation strength and individual coefficients. To predict tumor responses to immunotherapy, we employed the TIDE and subclass mapping models [30], with TIDE assessing immune evasion and subclass mapping gauging responses across different tumor subtypes. Results were visualized in a heatmap generated with the ‘pheatmap’ R package (version 1.0.12).

### 2.6. Development of a Prognostic Model for KIRC Using Wnt Pathway Genes

We initially analyzed the different expression of Wnt pathway genes between tumor and normal tissues. We identified genes with a prognostic value using univariate Cox regression analysis. These genes were further refined using LASSO regression to build a prognostic model. We stratified the samples into high-risk and low-risk groups based on median risk scores and assessed survival differences between these groups. The accuracy of our model was evaluated using ROC curves and AUC analysis with the “survivalROC” (version 1.0.3.1). Additionally, we integrated risk scores with clinicopathological features to construct a nomogram.

### 2.7. Cell Culture and RNA Interference

We acquired human renal cancer cell lines 786-O (RRID: CVCL_1051, Procell CL-0010) and ACHN (RRID: CVCL_1067, Procell CL-0021), along with normal kidney HK-2 (RRID: CVCL_0302, CL-0109) cells, from the Procell Life Science & Technology Co., Ltd. (Wuhan, China). ACHN and HK-2 cells were cultured in MEM (GIBCO, Waltham, MA, USA), while 786-O cells were maintained in RPMI-1640 (GIBCO, Waltham, MA, USA), both media supplemented with 10% fetal bovine serum. The cells were incubated at 37 °C in an atmosphere containing 5% CO_2_. We used siRNA from GenePharma (Shanghai, China) to downregulate *PLCB2* expression. The siRNA sequences were as follows: Sense (S): 5′-GGAGCCCAUUAUCACCCAUTT-3′ and Antisense (AS): 5′-AUGGGUGAUAAUGGGCUCCTT-3′. We transfected the cells at 80% confluence using a total concentration of 20 µM siRNAs, according to the GP-transfect-Mate reagent protocol. Proof of STR analysis for the human cell lines utilized in our research is provided in the Supplementary Files.

### 2.8. Reverse Transcription Quantitative PCR

Total RNA was extracted from cells using TRIZOL reagent (ABclonal, Wuhan, China). The extracted RNA was then reverse transcribed into cDNA using the Synthesis SuperMix protocol (TransGen, Beijing, China). We performed real-time quantitative PCR with Visual Green reagent (TransGen, Beijing, China). Primers for PLCB were custom-designed by SYNBIO (Suzhou, China). The sequences of the primers were as follows: Forward: 5′-GGAGCCCCATATCACCCACTT-3′, Reverse: 5′-ATGGGTGATAATGGGCTCCTT-3′. Data analysis was performed using the 2^−ΔΔCT^ method.

### 2.9. CCK-8 Cell Proliferation Assay

Cancer cells were plated at a density of 3000 cells per well in a 96-well plate (Biofil, Guangzhou, China). CCK-8 reagent (Beyotime, Shanghai, China), diluted 1:10 in a serum-free medium, and added to each well under conditions protected from light. After incubating for one hour, absorbance at 450 nm was measured using a spectrophotometer.

### 2.10. Colony Formation Assay

Gene-knockdown renal cancer cell lines were seeded at 800 cells per well in 6-well plates and cultured for 12 days, with medium changes every two days. Cells were washed twice with PBS, fixed with 4% paraformaldehyde, and stained with 0.1% crystal violet (Beyotime, Shanghai, China).

### 2.11. Wound Healing Assay

Cells were cultured in 6-well plates until they reached a 90% confluency. A wound was created using a 200 µL pipette tip, followed by washing with PBS to remove cellular debris. Serum-free medium was then added, and wound closure was monitored and photographed at 0 and 24 h using an inverted microscope (LEICACTR4000, Wetzlar, Germany).

### 2.12. Transwell Assay

For migration assays, 30,000 cells in 200 µL of the serum-free medium were placed in the upper chamber of an 8 µm pore Transwell (Biofil, Guangzhou, China), with complete medium in the lower chamber. For invasion assays, the Transwell membrane was coated with Matrigel (Corning, NY, USA), and 20,000 cells were seeded in the upper chamber. After 48 h of incubation, cells were washed with PBS, stained with crystal violet, and imaged. Cells on the underside of the membrane were quantified using ImageJ (version.win64).

### 2.13. Immunocytochemistry

786-O and ACHN cells were fixed with 4% formaldehyde, permeabilized with 0.5% Triton X-100 for 15 min, and washed with PBS. After blocking in 1% BSA for 30 min at room temperature, cells were incubated overnight at 4 °C with diluted *PLCB2* primary antibody. They were then incubated for one hour, with a fluorescently labeled secondary antibody (ABclonal, Wuhan, China), in the dark, followed by DAPI (SEVENBIO, Beijing, China) staining for 5 min. Images were captured using fluorescence microscopy.

### 2.14. Bulk RNA-Seq

The human renal carcinoma cell line 786-O was divided into two groups: one with reduced expression of the PLCB2 gene through targeted siRNA, and the other transfected with non-specific siRNA serving as a control. Total RNA was extracted using Trizol (ABclonal, Wuhan, China) and subjected to paired-end sequencing on an Illumina PE250 platform. Quality control was performed using fastp software (version, 0.23.4), with subsequent alignment to the reference genome using HISAT2 and gene expression quantification with Stringtie. Differential expression analysis was conducted using edgeR (version, 3.38) software, and gene involvement in biological pathways was analyzed through KEGG and GO enrichment analyses.

### 2.15. Western Blotting

Total protein was extracted using a commercial protein extraction kit (Seven, Beijing, China). Protein concentration was determined using the BCA Protein Assay Kit (Beyotime, Shanghai, China). Twenty micrograms of protein per sample were subjected to SDS-PAGE and transferred to PVDF membranes. The membranes were blocked with 5% non-fat milk to prevent non-specific binding and incubated overnight at 4 °C with the primary antibody. This was followed by a one-hour incubation at room temperature with the secondary antibody. Antibody information in Supplementary Table S1 Protein detection was performed using a SuperSignal chemiluminescent substrate (EPIZYME, Shanghai, China).

### 2.16. Statistical Analysis

We compared the gene expression between normal KIRC and tumor samples using one-way ANOVA. For comparisons between the two groups, we used an independent *t*-test to identify statistically significant differences. A one-way analysis of variance (ANOVA) or Kruskal–Wallis test of variance was used to compare three or more groups. Each experiment was repeated at least three times, with data presented as mean ± standard deviation. Statistical significance was defined as *p* < 0.05. Data visualization and statistical analyses were performed using R software (v4.3.2) and GraphPad Prism (version 9.5).

## 3. Results

### 3.1. Comprehensive Profiling of CNV and SNV Patterns in Wnt Pathway Genes Across Diverse Cancer Types

**Figure 1:**
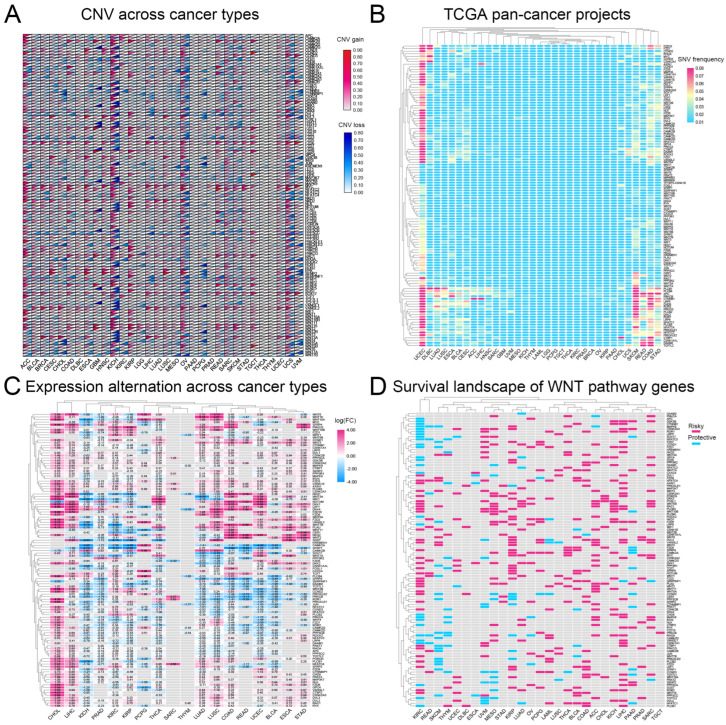
A comprehensive analysis of genetic changes in Wnt pathway genes across 32 types of tumors: (**A**) copy number variations are shown where red indicates an increase and blue a decrease, (**B**) the frequency of single nucleotide variations, with red showing higher occurrences and blue lower ones, (**C**) the changes in gene expression, represented through a color scale that reflects log2 fold changes, and (**D**) the heatmap showing the impact of these genes on survival, with red highlighting risk-enhancing genes, blue indicating protective genes, and gray showing genes without significant survival correlations.

We analyzed CNVs and SNVs in 117 Wnt-signaling pathway genes across 32 cancer types. Our results, depicted in Figure 1A,B, indicate significant heterogeneity in CNVs and SNVs among these genes. Notably, the kidney chromophobe (KICH) exhibited distinct CNV profiles with pronounced alterations, whereas the thyroid carcinoma (THCA) and thymoma (THYM) showed fewer changes, suggesting unique genetic patterns. Furthermore, the uterine corpus endometrial carcinoma (UCEC) and the skin cutaneous melanoma (SKCM) displayed high frequencies of SNVs. A heatmap (Figure 1C) based on log2-fold change values demonstrated general overexpression of Wnt pathway genes in cancers, except for genes such as *CAMK2A*, *SFRP1*, and *SOX17*, which were underexpressed in several tumor types. This study underscores the complex role of Wnt pathway genes in cancer and highlights potential targets for therapeutic intervention.

### 3.2. Most Wnt-Related Genes Are Risk Factors for Tumourigenesis

Research indicates that abnormal activation of the Wnt-signaling pathway is closely linked to the development of various cancers [31,32]. Based on this, we analyzed the correlation between the Wnt pathway gene expression in tumors and patient survival. We classified genes as ‘risk’ or ‘protective’ based on their impact on survival rates. High expression of protective genes was associated with improved survival. In contrast, elevated expression of risk genes correlated with worse outcomes. Our analysis revealed that most Wnt pathway genes predominantly act as risk factors in the progression of KIRC (Figure 1D).

### 3.3. Cluster Analysis of Wnt-Signaling Pathway Activity in KIRC

**Figure 2:**
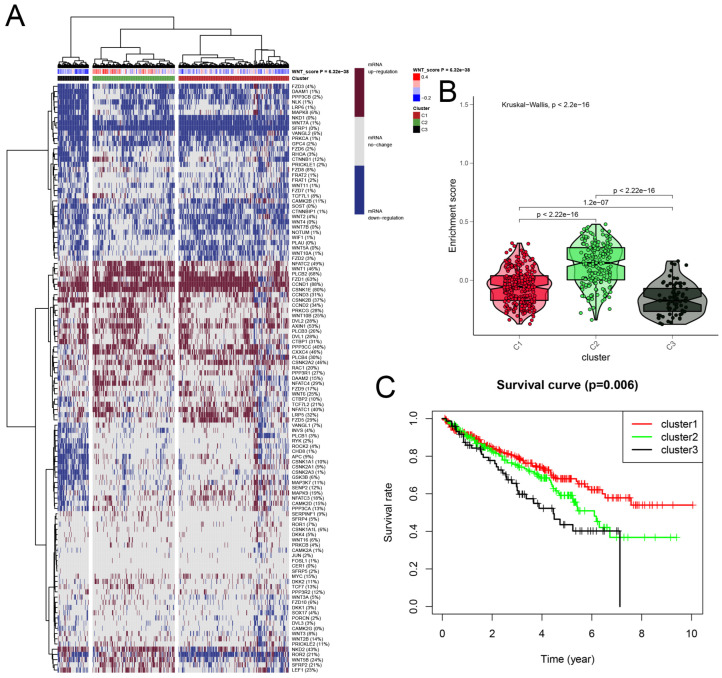
A detailed cluster analysis of tumor samples from the TCGA database, focusing on the differential expression of Wnt pathway genes: (**A**) delineates gene expression profiles within three distinct clusters: Cluster 1 exhibits up-regulation, Cluster 3 shows down-regulation, and Cluster 2 maintains stable gene expression levels, each correlated with specific Wnt scores; (**B**) utilizes a violin plot to quantitatively assess the enrichment scores across these clusters, providing insights into their statistical distributions and variances; (**C**) plots Kaplan-Meier survival curves, comparing survival outcomes across the clusters, with the highest survival rates observed in Cluster 1, moderate in Cluster 2, and the lowest in Cluster 3.

We developed a Wnt scoring model to assess the roles of Wnt pathway genes in RCC based on their mRNA expression profiles. The model categorized KIRC samples into three clusters: Cluster 1 with high Wnt activity, Cluster 2 with normal activity, and Cluster 3 with reduced activity (Figure 2A). Differences in Wnt activity across these clusters were visually represented in a violin plot (Figure 2B). Survival analysis showed that patients in Cluster 1, with higher Wnt activity, had a median survival rate exceeding 50%. This was significantly higher than survival rates in Cluster 2. Patients in Cluster 3, with the lowest Wnt activity, showed the poorest survival outcomes (Figure 2C). These findings suggest that Wnt scores could serve as a significant prognostic indicator in KIRC, potentially guiding therapeutic strategies.

### 3.4. Drug Sensitivity Analysis and Identification of Potential Therapeutic Agents Through Wnt Pathway Profiling in Cancer

**Figure 3:**
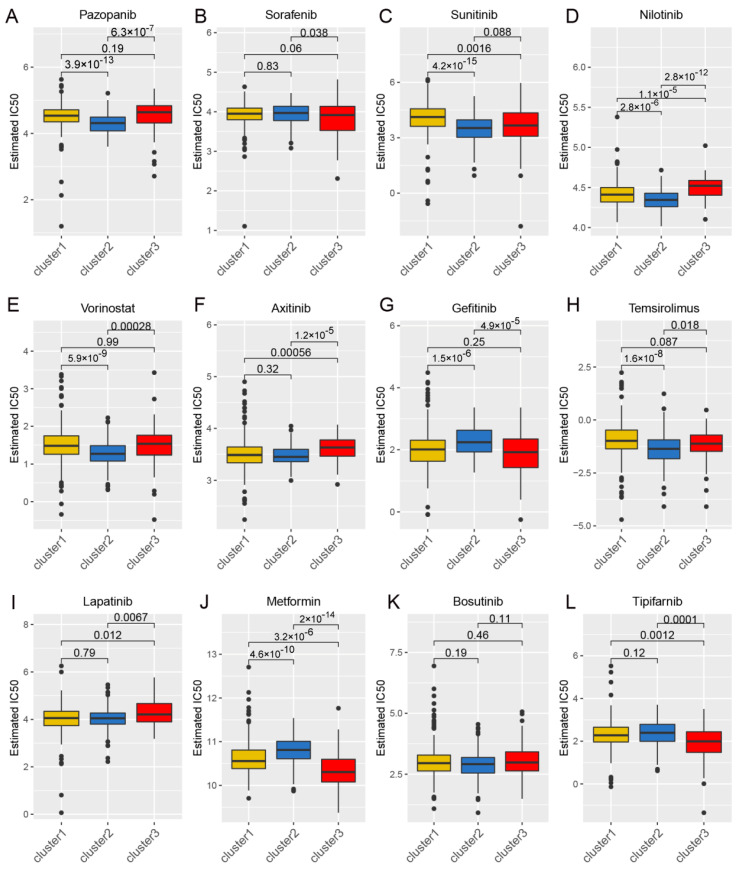
The correlation between gene expression clusters in the Wnt pathway and their responsiveness to chemotherapy. Panels (**A**–**L**) provide detailed half-maximal inhibitory concentration (IC50) values for three distinct gene expression clusters in response to a series of 12 chemotherapeutic agents. The agents examined include pazopanib, sorafenib, sunitinib, nilotinib, vorinostat, axitinib, gefitinib, temsirolimus, lapatinib, metformin, bosutinib, and tipifarnib.

**Figure 4:**
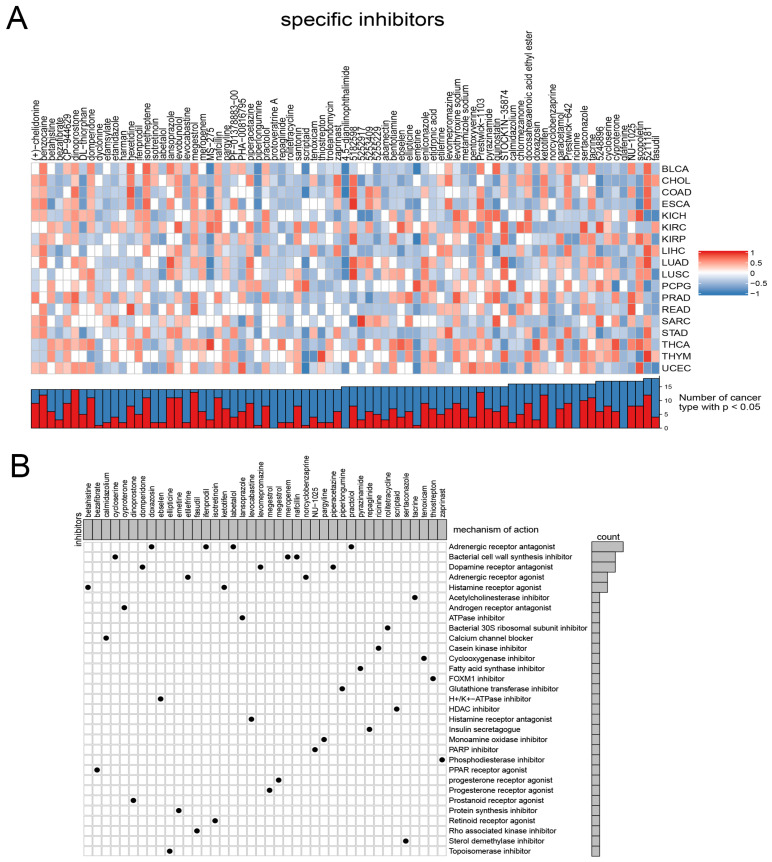
Presents the results from the CMap analysis. (**A**) illustrates the enrichment profiles of various compounds across tumor samples, highlighting the specific associations between distinct compounds and tumor types. (**B**) investigates the proposed mechanisms of action for these compounds.

Considering the continued prominence of chemotherapy as an auxiliary treatment in clinical practice, our study aimed to identify compounds with varying sensitivity based on Wnt scores in cancer patients. Utilizing ridge regression analysis to handle multicollinearity among predictors, we analyzed data from the GDSC database, identifying 12 drugs with differential sensitivity across Wnt score-defined patient groups (Figure 3). The selected drugs include first- and second-line therapies for RCC, such as pazopanib, sorafenib, sunitinib, axitinib, and temsirolimus, which are widely recognized for their roles in RCC treatment [33,34,35]. Drugs such as Pazopanib and Lapatinib showed increased cytotoxicity in the order of C3 > C1 > C2. In contrast, compounds like Sorafenib and Sunitinib were more effective in cluster C1 > C3. Additionally, analysis of the CMap database revealed 79 compounds targeting Wnt-related genes, with detailed assessments of 40 such compounds presented in Figure 4 and their mechanisms documented in Supplementary Material Table S2. Our study not only aids in guiding clinical drug use but also improves our understanding of the interactions between drugs and genes, helping to advance the development of targeted therapies based on molecular profiling.

### 3.5. Interplay of Wnt Pathway Genes with Oncogenes, HDACs, and SIRT Family Proteins in Gene Expression Regulation

**Figure 5:**
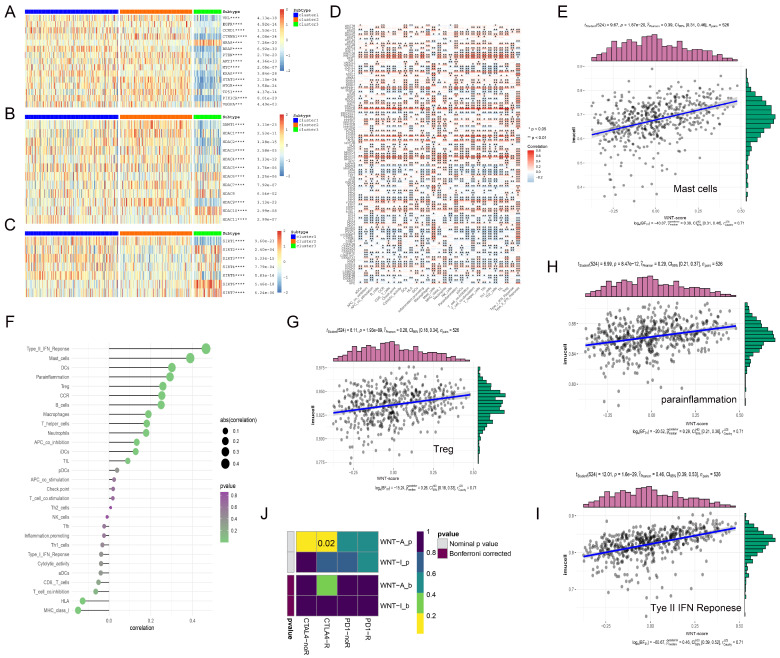
The complex interactions between Wnt pathway genes and various regulatory factors in RCC. (**A**–**C**) display heatmaps illustrating the correlations between Wnt pathway genes and three key gene families: traditional oncogenes (**A**), HDAC family genes (**B**), and Sirtuin family genes (**C**). (**D**) presents a heatmap detailing the correlations between immune infiltration-related factors and Wnt pathway genes, with red indicating positive correlations and gray indicating negative correlations; significance levels are denoted by asterisks. (**E**,**G**–**I**) feature scatterplots that demonstrate positive correlations between four specific immune infiltration factors and Wnt scores. (**F**) shows the strengths of these correlations, with the intensity of color reflecting *p*-values and the size of spheres representing the magnitude of correlation. (**J**) explores the differential response to CTLA-4 and PD-1 inhibitors between Wnt active and inactive groups using subclass mapping analysis, revealing a markedly higher sensitivity to CTLA-4 inhibitors in the Wnt active group, with a statistically significant *p*-value of 0.02. * *p* < 0.05, ** *p* < 0.01, **** *p* < 0.0001.

We systematically analyzed the interactions between Wnt pathway genes and classical oncogenes across three distinct sample groups, utilizing hierarchical clustering to visualize our findings (Figure 5). Cluster 3, with low Wnt-signaling activity, showed significant upregulation of the oncogene *HRAS*. Tumor suppressor genes such as *TP53*, *PTEN*, and *VHL* were downregulated in this cluster (Figure 5A). We also investigated the role of histone deacetylases (HDACs), which remove acetyl groups from histones, leading to chromatin condensation and gene expression suppression. Notably, *HDAC6* and *HDAC10* were abnormally expressed in Cluster 3. In Cluster 1, with high Wnt-signaling activity, these enzymes were significantly downregulated (Figure 5B). Furthermore, we explored the SIRT family proteins’ roles, particularly noting that the tumor suppressor gene *SIRT3* was highly expressed in Cluster 1, whereas *SIRT2*, *SIRT6*, and *SIRT7* expressions were reduced in the same cluster (Figure 5C). These observations highlight the complex interactions between Wnt pathway genes and key epigenetic regulatory factors, pointing to their potential impact on cancer progression.

### 3.6. Exploring the Impact of Wnt Pathway Activation on Immune Cell Infiltration and Immunotherapy Efficacy in Cancer

In our study, we examined the correlation between genes in the Wnt-signaling pathway and immune cell infiltration within the tumor microenvironment (TME), which includes tumor cells, immune cells, and various other components, pivotal in cancer progression and immune evasion [36]. In the analysis, we observed that genes such as *WNT10A* and *PLCB2* exhibited strong positive correlations with immune cell infiltration (Figure 5D). Specifically, *WNT10A* was significantly associated with T cell-related functions, including T cell co-stimulation and TIL, highlighting its potential role in promoting T cell-mediated immunity. Similarly, *PLCB2* demonstrated high correlations with T helper cells and immune checkpoint molecules, suggesting its involvement in the modulation of adaptive immune responses. Both genes were positively associated with inflammation-promoting markers and Type I IFN response, while negatively correlated with Mast cells and Type II IFN response, reflecting their distinct but complementary roles in orchestrating the immune landscape of the TME. In contrast, *DAAM1* and *CTBP2* exhibited significant negative correlations with immune cell infiltration. Notably, elevated Wnt scores were associated with higher levels of immune markers, including mast cells, which are known for their role in releasing histamine and other mediators to regulate inflammation, and Treg cells, a subset of T cells that maintain immune tolerance by suppressing excessive immune responses (Figure 5E,G–I). Conversely, HLA, a critical component of the major histocompatibility complex that presents antigens to T cells for immune recognition, showed a negative correlation with Wnt scores (Figure 5F).

In the context of cancer therapy, where immune checkpoint inhibitors, such as ipilimumab, nivolumab, and pembrolizumab, have proven effective [37,38], we observed that Wnt pathway activity significantly correlated with CTLA-4 expression, a key marker of response to these therapies. TIDE analysis confirmed that KIRC patients with high Wnt pathway gene expression were more responsive to CTLA-4 targeted therapy (Figure 5J). This suggests that renal cancer patients with higher Wnt scores might particularly benefit from CTLA-4 targeted treatments, potentially leading to improved survival outcomes.

### 3.7. Prediction Model Based on LASSO Regression

**Figure 6:**
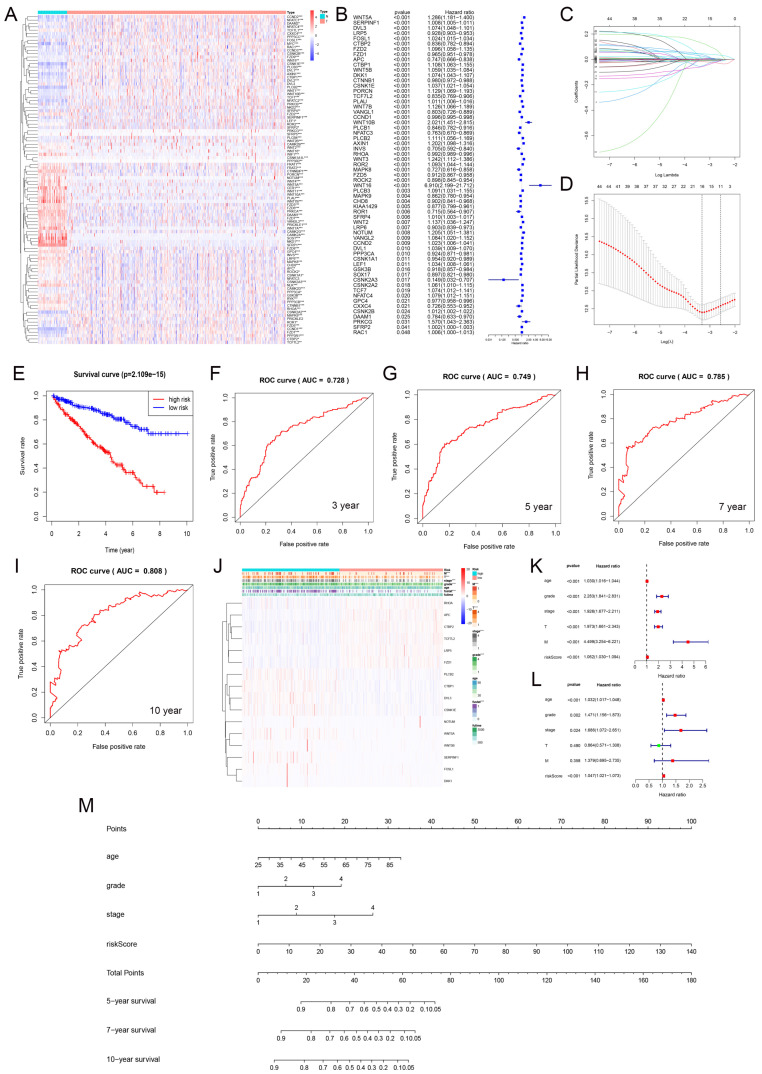
The gene expression and prognosis in the Wnt pathway. (**A**) shows differential expression of genes between tumor and normal samples. (**B**) details a hazard ratio analysis for 33 significant genes. (**C**,**D**) describe the selection of 16 key genes using LASSO regression. (**E**) contrasts survival outcomes between low-risk and high-risk groups. (**F**–**I**) display ROC curves for 3, 5, 7, and 10-year predictions. (**J**) correlates these genes with clinical-pathological features. (**K**,**L**) present univariate and multivariate Cox regression analyses. (**M**) introduces a nomogram integrating multiple prognostic indicators into a predictive model. * *p* < 0.05, ** *p* < 0.01, *** *p* < 0.001.

We analyzed the differential expression of Wnt pathway-related genes in normal and tumor samples from the TCGA-KIRC dataset (Figure 6A). Hazard ratio analysis identified 33 Wnt pathway genes that were significantly associated with KIRC progression (Figure 6B). We then applied LASSO regression to select 16 prognostic marker genes, including *RHOA*, *APC*, and *PLCB2* among others, and developed a prognostic model (Figure 6C,D). The model stratified KIRC samples into high-risk and low-risk groups using median risk scores. Patients in the low-risk group had significantly better survival rates (Figure 6E). We validated the model’s accuracy with ROC curve analysis. The AUC values for predicting 3-, 5-, 7-, and 10-year survival were 0.728, 0.749, 0.785, and 0.808, respectively (Figure 6F–I). These results demonstrated strong predictive reliability. Furthermore, our model significantly correlated with essential clinical parameters such as tumor grade, stage, and metastasis in KIRC (Figure 6J).

### 3.8. Prognostic Evaluation of KIRC Patients Using a Nomogram

We carried out univariate and multivariate Cox regression analyses to evaluate the prognostic value of Wnt risk scores together with clinical characteristics such as age, tumor grade, and stage for patients with RCC. The results (Figure 6K,L) showed that Wnt scores are an independent prognostic factor in KIRC. Using these findings, we developed a nomogram (Figure 6M) to visually predict patient outcomes. This tool integrates multiple prognostic variables to provide clinicians with a quantitative method to assess patient prognosis, potentially guiding personalized treatment strategies.

### 3.9. Elevated PLCB2 Expression as a Marker of Poor Prognosis in Renal Cell Carcinoma

**Figure 7:**
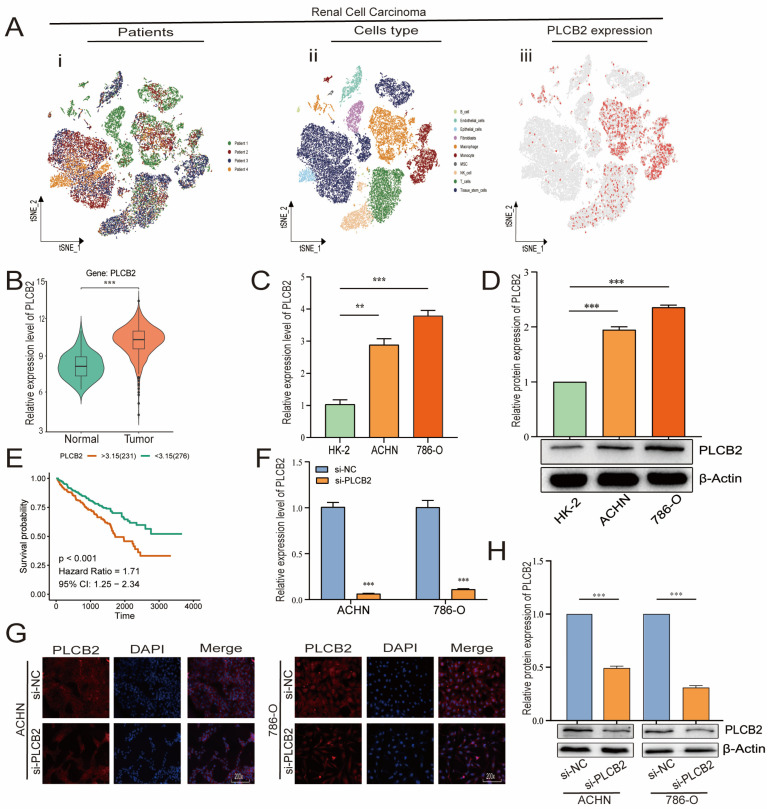
*PLCB2* expression analysis. (**A**) Clustering, annotation, and expression of the PLCB2 gene in all RCC samples from the scRNA-seq dataset GSE152938. (**B**) Examination of *PLCB2* mRNA levels in KIRC samples sourced from the TCGA database. (**C**,**D**) Demonstration of the use of qRT-PCR and Western blot to analyze *PLCB2* mRNA and protein levels in normal kidney tissue and renal cancer cell lines. (**E**) Presentation of a survival analysis that correlates *PLCB2* expression levels with patient outcomes in KIRC. (**F**–**H**) qRT-PCR, immunofluorescence, and Western blot analyses of mRNA and protein expression levels of si-NC and si-*PLCB2* in RCC lines. ** *p* < 0.01, *** *p* < 0.001.

We focused our research on the expression of *PLCB2*, a key prognostic gene in RCC, as detailed in Figure 7A,B. scRNA-seq data showed that *PLCB2* is expressed in multiple cell clusters across renal tumor samples. Furthermore, survival data from the TCGA database revealed that high *PLCB2* expression is significantly associated with poor prognosis (Figure 7E). To further investigate this association, we conducted qRT-PCR and Western blot analyses, which showed significantly higher levels of *PLCB2* in RCC cell lines (ACHN, 786-O) compared to normal renal epithelial cells (Figure 7C,D). These findings suggest a critical role for *PLCB2* in promoting tumor progression.

### 3.10. PLCB2 Depletion Inhibits Proliferation and Migration of RCC

**Figure 8:**
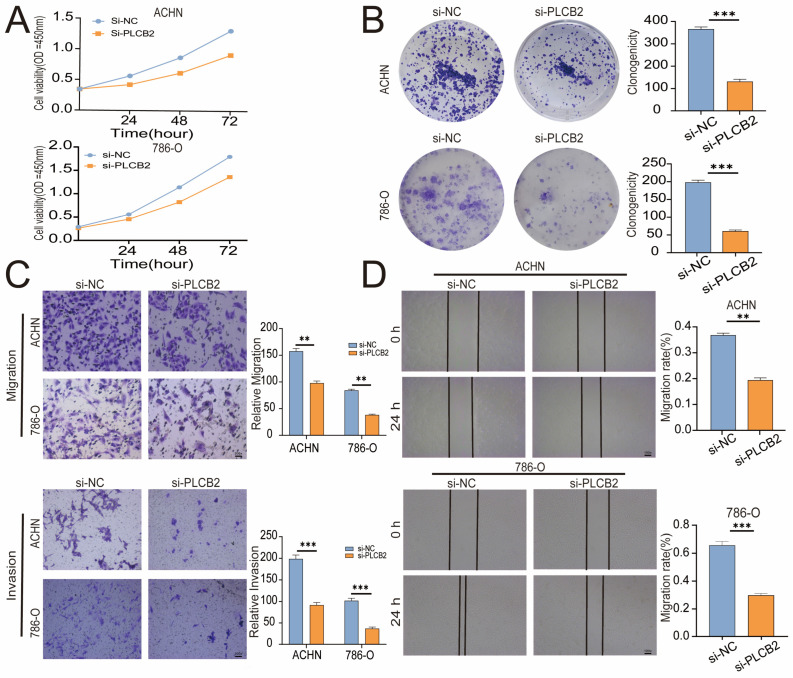
Effects of *PLCB2* Knockdown on RCC Behaviors. (**A**,**B**) Cell proliferation in renal cancer cells with *PLCB2* knockdown and control groups, assessed via CCK-8 and colony formation assays. (**C**,**D**) Migration and invasion capabilities of the *PLCB2* knockdown and control groups measured using scratch and transwell assays. ** *p* < 0.01, *** *p* < 0.001.

To explore the biological roles of *PLCB2* in RCC, we used siRNA to reduce PLCB2 gene expression in the 786-O and ACHN cell lines. We used qRT-PCR, immunofluorescence, and Western blot analyses to confirm *PLCB2* reduction. All methods showed significant decreases in *PLCB2* mRNA and protein levels (Figure 7F–H). Cell proliferation and colony formation assays showed that reducing the *PLCB2* expression significantly hindered both the proliferation, and the colony-forming capabilities compared to controls. Furthermore, scratch assays indicated that reduced *PLCB2* expression markedly decreased the migration abilities of these cells (Figure 8A,B,D). These findings underscore the crucial role of *PLCB2* in promoting the proliferation and migration of RCC cells.

### 3.11. PLCB2 Induces EMT to Enhance Migration and Invasion of RCC Cells

**Figure 9:**
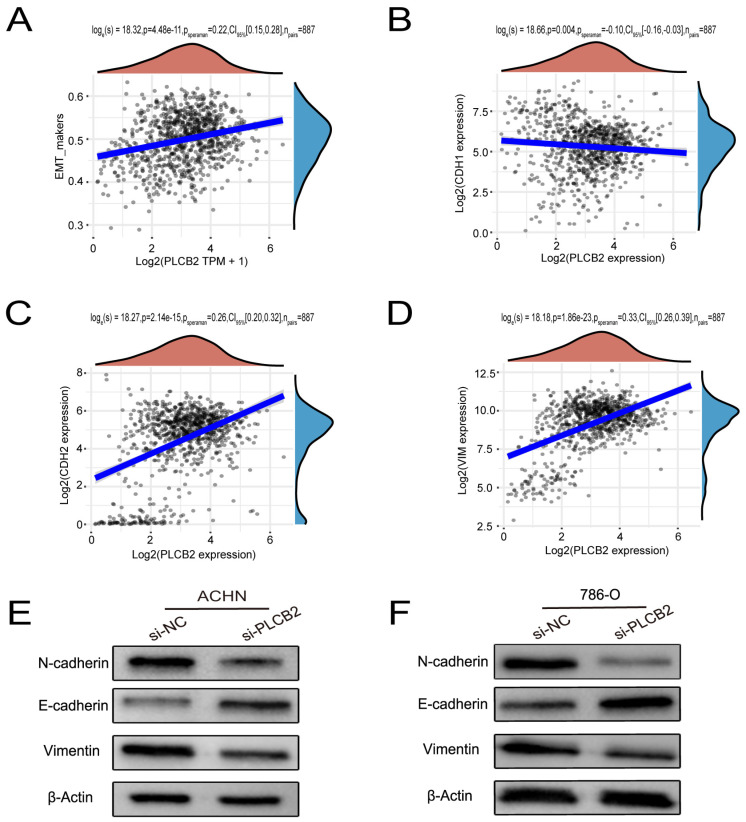
*PLCB2*’s Role in Enhancing EMT in RCC. (**A**–**D**) These figures present a correlation analysis between *PLCB2* mRNA expression and EMT markers—E-cadherin (CDH1), N-cadherin (CDH2), and Vimentin (VIM). (**E**,**F**) Western blot analyses assessing the levels of *PLCB2*, E-cadherin, N-cadherin, and Vimentin proteins in renal cancer cells with knockdown *PLCB2* compared to control groups.

Our findings suggest that a high expression of *PLCB2* may exacerbate the malignancy of RCC by promoting cell proliferation, migration, and invasion. We utilized Transwell assays to explore the functional role of *PLCB2* in RCC cell migration and invasion, revealing that a siRNA-mediated reduction in *PLCB2* significantly impairs these capabilities in renal cancer cells (Figure 8C). EMT is pivotal for metastasis and invasion of tumor cells. We examined the relationship between the *PLCB2* expression and key EMT biomarkers: CDH1 (E-cadherin), CDH2 (N-cadherin), and VIM (Vimentin). Our analyses identified a significant inverse correlation between *PLCB2* expression and CDH1 mRNA levels, while positive correlations were found with mesenchymal markers CDH2 and VIM mRNA levels (Figure 9A–D). Additionally, we used Western blot analysis to assess the impact of the *PLCB2* reduction on EMT marker protein levels. The results showed that a *PLCB2* depletion reduced N-cadherin and Vimentin levels while increasing E-cadherin levels (Figure 9E,F). These findings present *PLCB2* as a critical molecular regulator in the EMT process, significantly influencing the cellular dynamics essential for RCC progression.

### 3.12. Regulation of EMTby PLCB2 Through the PI3K/AKT Pathway in RCC

**Figure 10:**
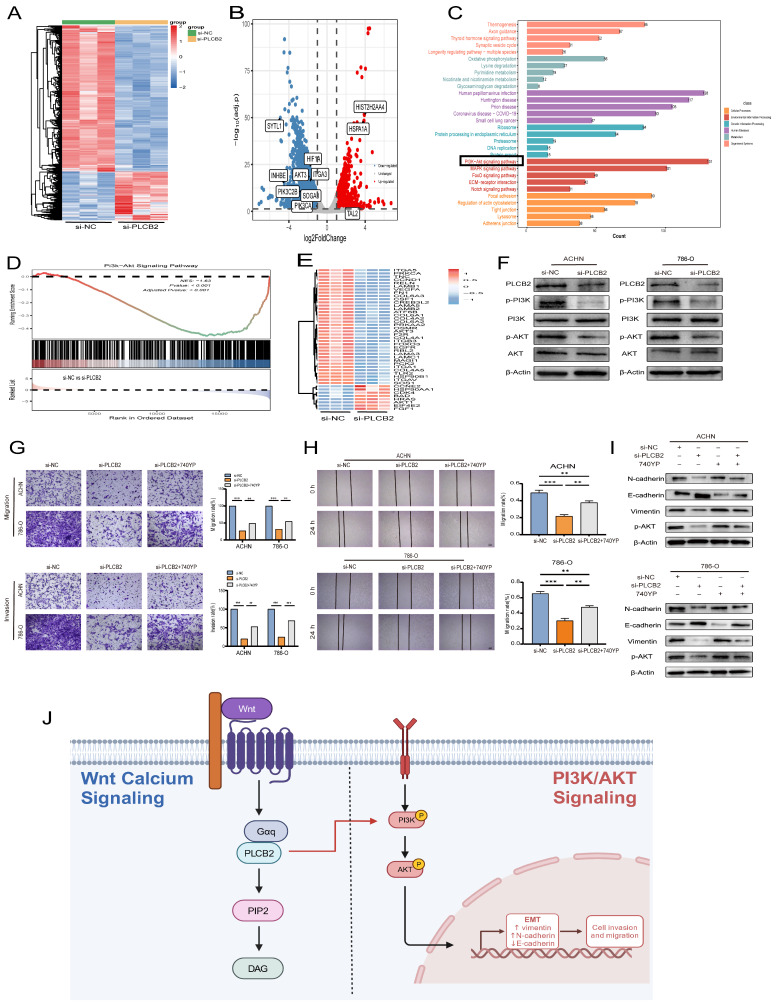
Regulation of EMT in RCC by *PLCB2* through the PI3K/AKT Pathway. (**A**,**B**) Heatmap and volcano plot display differentially expressed genes from RNA-seq analysis comparing groups with *PLCB2* knockdown to control groups. (**C**) Functional enrichment analysis of the differentially expressed genes is shown. (**D**) GSEA comparing the gene expression profiles of groups with *PLCB2* knockdown and controls. (**E**) Expression levels of genes related to the PI3K/AKT pathway in groups with *PLCB2* knockdown versus control groups. (**F**) Western blot analysis of the changes in PI3K/AKT pathway-related protein expression between groups with *PLCB2* knockdown and controls. (**G**) Transwell assays assessing the impact of *PLCB2* knockdown and 740YP treatment on RCC cell invasion and migration. (**H**) Scratch assays evaluating the effect of *PLCB2* knockdown and 740YP treatment on RCC cell migration. (**I**) Western blot analysis determining changes in the protein levels of E-cadherin, N-cadherin, Vimentin, and p-AKT following *PLCB2* knockdown and 740YP treatment. (**J**) Diagram of the mechanism of *PLCB2* in RCC. *PLCB2* in the Wnt/calcium signaling regulates RCC invasion, metastasis, and the EMT process through the activation of the PI3K/AKT signaling pathway. ** *p* < 0.01, *** *p* < 0.001.

We explored the molecular mechanisms by which the *PLCB2* influences EMT in RCC. RNA-seq analysis identified 5112 differentially expressed genes in 786-O RCC cells after siRNA-mediated *PLCB2* reduction. This included 1277 upregulated and 3835 downregulated genes (Figure 10A,B). Functional enrichment analysis indicated significant involvement of these genes in EMT-associated processes, particularly in tight junction, focal adhesion and adherence junction. Further KEGG pathway analysis implicated *PLCB2* in the regulation of EMT via the PI3K/AKT signaling pathway (Figure 10C,D). Figure 10E illustrates the changes in the expression levels of genes associated with the PI3K/AKT signaling pathway following the downregulation of *PLCB2*. Supporting this, Western blot analysis showed a significant decrease in the phosphorylation levels of PI3K and AKT after reducing *PLCB2* levels (Figure 10F), suggesting that *PLCB2* may modulate EMT in RCC through the activation of the PI3K/AKT signaling pathway.

To further validate whether the activation of the PI3K/AKT pathway is crucial for *PLCB2*-mediated EMT, we treated *PLCB2* knockdown RCC cells with the PI3K/AKT pathway activator 740Y-P (20 μM). The results showed that the addition of 740Y-P significantly restored the invasion and migration abilities that were lost in *PLCB2* knockdown cells (Figure 10G,H). Additionally, Western blot analysis indicated that adding 740Y-P to *PLCB2* knockdown cells partially rescued the expression of E-cadherin, N-cadherin, and Vimentin (Figure 10I). These findings suggest that *PLCB2* may regulate EMT in RCC cells through the activation of the PI3K/AKT pathway.

## 4. Discussion

RCC, the most prevalent kidney cancer among adults, has experienced a rising global incidence. Despite advances in targeted agents and immunotherapeutic strategies that have improved outcomes in certain patient subsets, the prognosis for patients with advanced or metastatic RCC remains limited, underscoring the persistent need for novel therapeutic targets and strategies [1,3]. The central role of EMT in driving tumor invasion and metastasis in RCC is well-recognized [39,40,41,42]. For instance, TIMP1 promotes RCC metastasis by inducing EMT [40], while CSN5 facilitates tumor progression through the stabilization of ZEB1, thereby enhancing EMT-mediated tumor spread [41]. Additionally, MEF2A has been reported to repress EMT and thereby inhibit RCC invasion and migration by modulating the Wnt/β-catenin signaling pathway [42]. Collectively, these findings indicate that targeting EMT and its associated molecular pathways remains a promising strategy in RCC therapy research.

Our investigation reveals a pivotal role for *PLCB2* in governing EMT in RCC through the activation of the PI3K/AKT signaling pathway (Figure 10J). The PI3K/AKT cascade, a fundamental regulator of cell proliferation, survival, and metabolism, is integral to EMT and tumorigenesis in diverse cancers, including RCC [43,44,45,46]. Prior studies have shown that TGFBI enhances both EMT and proliferation via the PI3K/AKT/mTOR/HIF-1α pathway [47], and MUC15 promotes RCC metastasis by elevating phosphorylated AKT levels and increasing MMP2/MMP9 expression [48]. Our study enhances the understanding of tumor biology by emphasizing the crucial role of the PI3K/AKT pathway in driving EMT-related malignant behaviors in RCC. In addition, a recent study by Hwang et al. also demonstrated the critical role of the PI3K/AKT pathway in RCC, which echoes our findings [49].

Although *PLCB2* has not been widely studied in RCC, emerging evidence indicates its oncogenic relevance in several tumor types. The downregulation of *PLCB2* expression in melanoma reduces cell viability and induces apoptosis by altering the Ras/Raf/MAPK pathway [6]. Additionally, extracts from Astragalus mongholicus Bunge can modulate pyroptosis in colonic epithelial cells by influencing *PLCB2* expression [50]. In RCC, consistently with our findings, Wu et al. observed a marked elevation in *PLCB2* mRNA levels in tumor tissues, compared to their normal counterparts [51]. Our findings position *PLCB2* as a potential oncogene and a promising therapeutic target that warrants further exploration in RCC.

Notably, therapeutic strategies aimed at modulating the PI3K/AKT axis are emerging as promising approaches in RCC management. Pre-clinical and early-phase clinical studies have explored the efficacy of PI3K or AKT inhibitors, either alone or in combination with immunotherapies or VEGF-targeted agents, to enhance anti-tumor responses and overcome resistance [52,53]. Our study suggests that interventions targeting *PLCB2* may complement these strategies, potentially improving the prognosis of patients with RCC.

While the interaction between the Wnt pathway and the PI3K/AKT pathway is well-established, its crosstalk with other key intracellular signaling pathways, such as MAPK and Notch, warrants further investigation to better understand RCC progression. The MAPK pathway, a critical regulator of cell growth and survival, is frequently dysregulated in cancers, including RCC [54]. Wnt signaling can activate the MAPK pathway by inducing receptor tyrosine kinases (e.g., EGFR), leading to a downstream phosphorylation of MAPK components like ERK1/2. Conversely, MAPK signaling enhances β-catenin transcriptional activity, amplifying Wnt-driven gene expression and promoting tumor proliferation and metastasis [55,56]. Similarly, the Notch pathway, a pivotal regulator of cell fate, intersects with Wnt signaling by upregulating the Notch receptors and stabilizing β-catenin, facilitating nuclear localization and transcriptional activation. This synergy supports cancer stem cell maintenance, fueling tumorigenesis and therapy resistance [57,58]. The interactions between the Wnt, MAPK, and Notch pathways underscore the complex crosstalk between multiple signaling networks that drive tumor progression, highlighting the need for further investigation into their underlying molecular mechanisms.

Nevertheless, certain limitations must be acknowledged. First, this study primarily used in vitro models, including HK-2, 786-O, and ACHN cell lines, which cannot fully replicate the complexity of RCC or its tumor microenvironment. Second, the TCGA-KIRC dataset used in this study, while providing valuable genomic insights, lacks clinical samples and in vivo model validation, further limiting the translational potential of the findings. Third, this study did not investigate whether targeting *PLCB2* could enhance the effectiveness of current treatments, such as tyrosine kinase inhibitors or immune checkpoint inhibitors. Further research is needed to determine the role of *PLCB2* inhibition in RCC treatment. Future studies should focus on using clinical samples and animal models, along with additional experiments, to investigate the interactions between *PLCB2* and the Wnt pathway, its combined effects with current therapies, and its potential to improve treatment strategies.

Targeted therapeutic drugs like sorafenib, sunitinib, and pazopanib have shown efficacy in RCC treatment, but are often limited by side effects such as hypertension and gastrointestinal issues [59]. Our study highlights *PLCB2* as a potential alternative target and provides a foundation for developing *PLCB2*-specific inhibitors. Current evidence indicates that small-molecule inhibitors represent a viable approach, with high-throughput screening and molecular optimization offering the potential to develop drugs that are both highly selective and exhibit minimal side effects. Additionally, based on our study of *PLCB2*, combination therapies targeting *PLCB2* alongside related molecules, such as PI3K/AKT, may further enhance therapeutic efficacy. However, significant challenges remain in translating these strategies into clinical applications. For instance, issues related to drug delivery efficiency and tissue specificity, particularly in central nervous system disorders, persist, with the blood–brain barrier representing a major obstacle. Furthermore, given *PLCB2*’s widespread involvement in neural signaling and immune regulation, systemic inhibition may lead to adverse effects such as neurotoxicity or immune dysfunction. Addressing these concerns will require careful optimization of drug design and dosing strategies.

In conclusion, our study identifies *PLCB2* as a significant regulator of EMT in RCC through its activation of the PI3K/AKT pathway, thereby promoting tumor invasion and metastasis. We highlight *PLCB2*’s emerging role in RCC biology and underscore its potential as a therapeutic target. As we advance our understanding of *PLCB2*’s molecular function and its involvement in signaling crosstalk, efforts to design novel interventions may ultimately improve the clinical management of advanced RCC.

## 5. Conclusions

In summary, our study demonstrates that elevated *PLCB2* expression in RCC significantly promotes tumor cell proliferation, EMT, and enhanced invasiveness. Importantly, the downregulation of *PLCB2* expression was found to inhibit these malignant phenotypes, underscoring its pivotal role in RCC metastasis through the PI3K/AKT signaling pathway. Consequently, *PLCB2* emerges as a critical prognostic marker and a promising therapeutic target in the management of RCC.

## Data Availability

The datasets used and/or analyzed during the current study are available from the corresponding author on reasonable request.

## Supplementary Materials

The following supporting information can be downloaded at: https://www.mdpi.com/article/10.3390/biomedicines13020304/s1, Document S1: STR analysis; Table S1: Primary Antibody Information; Table S2: Inhibitors and their mechanisms of action.

## Abbreviations

RCC	Renal cell carcinoma
EMT	epithelial-mesenchymal transition
*PLCB2*	Phospholipase C Beta 2
KIRC	Kidney renal clear cell carcinoma
qRT-PCR	quantitative real-time-polymerase chain reaction
CNV	copy number variation
SNV	single nucleotide variant
TCGA	The Cancer Genome Atlas
scRNA-seq	single-cell RNA sequencing
siRNA	small interfering RNA
PVDF	polyvinylidene fluoride
SDS-PAGE	sodium dodecyl sulfate–polyacrylamide gel electrophoresis
KEGG	Kyoto Encyclopedia of Genes and Genomes
GEO	Gene Expression Omnibus
WB	western blot
