# Supplementary material for: Phospholipase C Beta 2 as a Key Regulator of Tumor Progression and Epithelial-Mesenchymal Transition via PI3K/AKT Signaling in Renal Cell Carcinoma
Source: Biomedicines. 2025 Jan 26;13(2):304. doi: 10.3390/biomedicines13020304 (PMC11853330; doi:10.3390/biomedicines13020304)
Supplement: Supplementary file 1 [file biomedicines-13-00304-s001.zip › STR analysis.pdf]

HK-2 细胞出库质检单

一、产品信息

1. 细胞名称：HK-2（人肾皮质近曲小管上皮细胞）
2. 细胞货号：CL-0109
3. 出库日期：2024-03-19

二、检测项目及结果

检测项目	检测结果	
生长特性（贴壁/悬浮）	贴壁	
细胞形态	上皮细胞样	
细胞密度	＞75%	
细胞纯度	——	
细胞总量	～1×10^6/Cells	
细胞活力	＞95%	
HIV-1	有☐	无☑
HBV	有☐	无☑
HCV	有☐	无☑
支原体	有☐	无☑
细菌	有☐	无☑
酵母	有☐	无☑
真菌	有☐	无☑

三、质检员及质检日期

质检员：[illegible]　　　　质检日期：2024-03-16

质量合格，准予放行

武汉普诺赛生命科技有限公司 质检专用章

附表1：细胞株HK-2的STR位点和Amelogenin位点的基因分型结果。

STR Loci	样品名称：PC-H2024010231	数据库名称：HK-2
Amelogenin	X	X
CSF1PO	13	13
D2S1338	17,25	17,25
D3S1358	16	16,17
D5S818	12	12
D7S820	10,11	10,11
D8S1179	10,14	10,14
D13S317	9	9
D16S539	12	11,12
D18S51	12	12
D19S433	15,15.2	15,15.2
D21S11	28,30	28,30
FGA	20	20,22
PentaD	9,12	
PentaE	10,11	
TH01	9	9
TPOX	8,9	8,9
vWA	17,18	17,18
D6S1043	12,13	
D12S391	17.3,22	
D2S441	11,12	
ExPASy数据库匹配度93.62%，匹配位点数15（https://web.expasy.org/cellosaurus-str-search/）		

备注：

1. 根据国际细胞鉴定委员会(ICLAC)制定的细胞 STR 鉴定标准，细胞系的匹配度≥80% 时，认为它们具有相关性，即衍生于共同的祖先细胞；匹配度在 55% 至 80% 之间，需要进一步验证相关性；小于 55%，表明两者不具有相关性。
2. 图谱有效峰为真实的 PCR 条带;小峰和非特异性条带在计算中忽略不计。
3. STR 数据比对结果默认 ExPASy，数据来源包括 ATCC，DSMZ，JCRB 等细胞库以及文献和资料记载，数据库入口 https://web.expasy.org/cellosaurus-str-search/。

附图1：HK-2细胞(编号PC-H2024010231)STR位点和Amelogenin位点的基因分型结果。

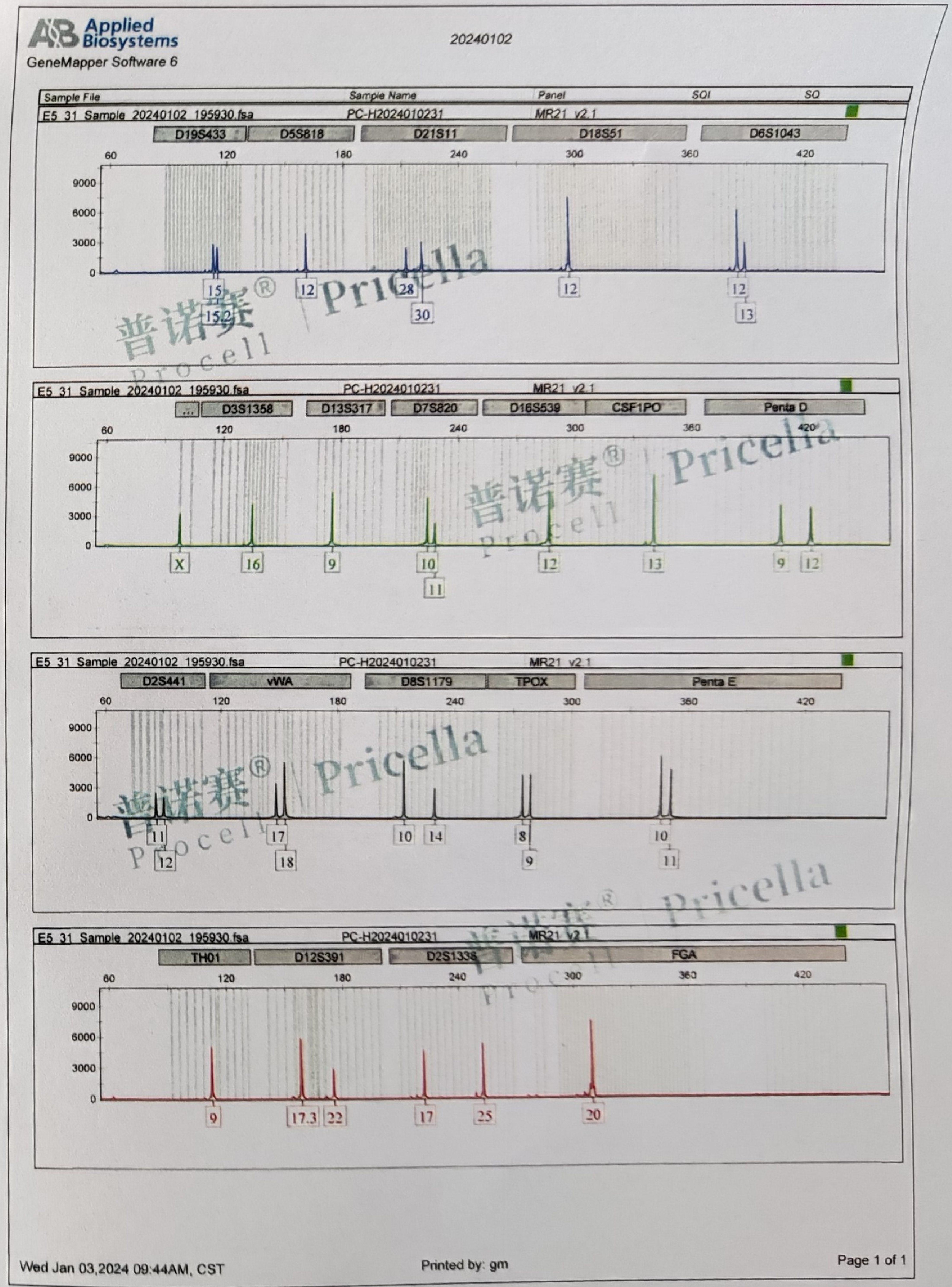

HK-2细胞STR鉴定报告

一、 材料处理和检验方法

取适量HK-2细胞(编号PC-H2024010231,1×10^6)使用TIANamp Genomic DNA Kit提取DNA，采用Microreader™21 ID System扩增20个STR位点和性别鉴定位点，使用智阅基因分析仪GenReader 7010进行PCR产物检测，使用GeneMapper Software6软件（Applied Biosystems）对检测结果进行分析，并与ExPASy数据库进行比对。

二、 检测结果

实验中阴性及阳性对照结果均正确。

HK-2 细胞株的 STR 位点和 Amelogenin 位点的基因分型结果见附表，分型图谱见附图。

三、 分析说明

HK-2 细胞株基因组 DNA 扩增后图谱清晰，分型结果良好。

四、 检验结论

1. HK-2 细胞株 DNA 进行细胞 STR 分型结果显示，细胞株中未发现人类细胞交叉污染。
2. 该细胞株 DNA 分型在细胞库中找到与其细胞分型 93.62%相匹配的细胞株，细胞株名称为 HK-2。

附图 1：786-O [786-0]细胞 STR 位点和 Amelogenin 位点的基因分型结果

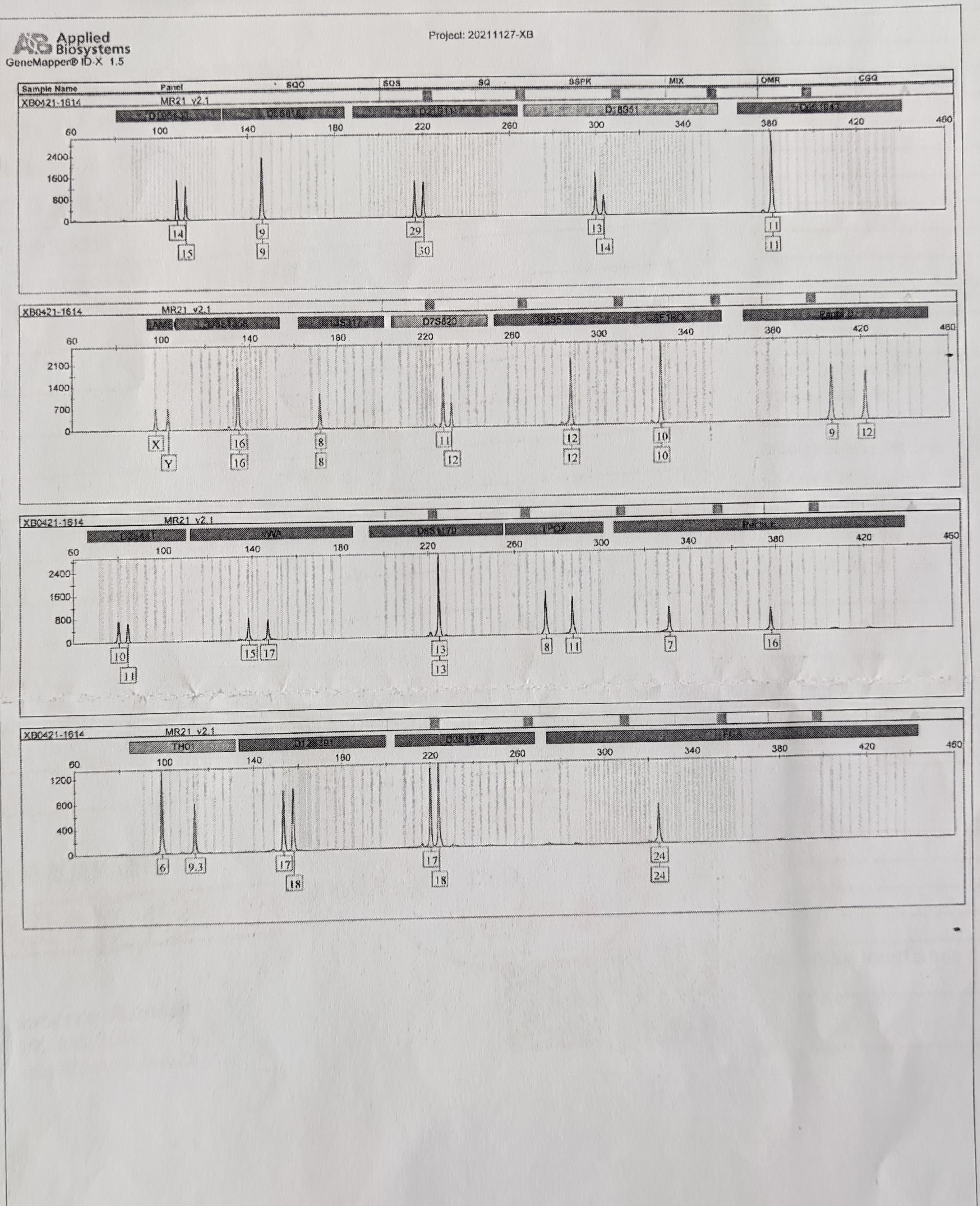

销售电话：400-650-3656
企业 QQ：4006503656
销售邮箱：sales@procell.com.cn
官方网站：www.procell.com.cn

附表 1：细胞株 786-O [786-0]的 STR 位点和 Amelogenin 位点的基因分型结果

STR Loci	样品名称：XB0421-1614	数据库名称：786-O [786-0]
Amelogenin	X,Y	X,Y
CSF1PO	10	10
D2S1338	17,18	17,18
D3S1358	16	16
D5S818	9	9
D7S820	11,12	11,12
D8S1179	13	13
D13S317	8	8
D16S539	12	12
D18S51	13,14	13,14
D19S433	14,15	14,15
D21S11	29,30	29,30
FGA	24	24
PentaD	9,12	9,12
PentaE	7,16	7,16
TH01	6,9.3	6,9.3
TPOX	8,11	8,11
vWA	15,17	15,17
D6S1043	11	
D12S391	17,18	
D2S441	10,11	
Cellosaurus 数据库匹配度 100.00%，匹配位点数 17（https://web.expasy.org/cellosaurus-str-search/）		

备注：

1. 根据国际细胞鉴定委员会(ICLAC)制定的细胞 STR 鉴定标准，细胞系的匹配度≥80%时，认为它们具有相关性，即衍生于共同的祖先细胞；匹配度在 55% 至 80% 之间，需要进一步验证相关性；小于 55%，表明两者不具有相关性。
2. 图谱有效峰为真实的 PCR 条带;小峰和非特异性条带在计算中忽略不计。
3. STR 数据比对结果默认 ExPASy，数据来源包括 ATCC，DSMZ，JCRB 等细胞库以及文献和资料记载，数据库入口 https://web.expasy.org/cellosaurus-str-search/。

Copyright ©2019-2020 Procell Life Science&Technology Co.,Ltd. All Rights Reserved

786-O [786-0]细胞 STR 鉴定报告

一、 材料处理和检验方法

取适量 **786-O [786-0]**细胞(编号 XB0421-1614,1×10^6)使用 Microread Genomic DNA Kit 提取 DNA，采用 Microreader™21 ID System 扩增 20 个 STR 位点和性别鉴定位点，使用 ABI 3130x1 型遗传分析仪进行 PCR 产物检测，使用 GeneMapperID-X 软件（Applied Biosystems）对检测结果进行分析，并与 ATCC、DSMZ、Cellosaurus 等数据库进行比对。

二、 检测结果

实验中阴性及阳性对照结果均正确。

786-O [786-0]细胞株的 STR 位点和 Amelogenin 位点的基因分型结果见附表，分型图谱见附图。

三、 分析说明

786-O [786-0]细胞株基因组 DNA 扩增后图谱清晰，分型结果良好。

四、 检验结论

1. **786-O [786-0]**细胞株 DNA 进行细胞 STR 分型结果显示，细胞株中未发现人类细胞交叉污染。
2. 该细胞株 DNA 分型在细胞库中找到与其细胞分型 100.00%相匹配的细胞株，细胞株名称为 **786-O [786-0]**。

Procell
www.procell.com.cn

销售电话：400-650-3656
企业 QQ：4006503656
销售邮箱： sales@procell.com.cn
官方网站： www.procell.com.cn

CL-0010

786-O [786-0]（人肾透明细胞腺癌细胞）

1.Origin and General Characteristics	
Cell Name	786-O
Synonyms	786-o; 786O; 786-0; 786.O; 786-O RCC; RCC 786-O; RCC_7860; RCC 7860; 7860; 786-0WT
Organism	Homo sapiens, Human
Age	58 years
Tissue	Kidney
Morphology	Epithelial
Growth Properties	Adherent
Descriptions	The cells display both microvilli and desmosomes, and can be grown in soft agar.<br>The cells produce a PTH like peptides that is identical to peptides produced by breast and lung tumors.<br>The peptide has an N terminal sequence similar to PTH, has PTH like activity, and has a molecular weight of 6000 daltons.
Biosafety Level	1
**2.Culture Conditions and Handling**	
Complete Growth Medium	RPMI-1640 (PM150110) + 10% FBS (164210-500) + 1% P/S (PB180120)
Subculturing	Remove and discard culture medium. Briefly rinse the cell layer with DPBS solution to remove all traces of serum that contains trypsin inhibitor.<br>Add 1.0 to 2.0 mL of Trypsin-EDTA solution to flask and observe cells under an inverted microscope until cell layer is dispersed (usually within 2~3min). Cells that are difficult to detach may be placed at 37℃ to facilitate dispersal.<br>Add 4.0 to 6.0 mL of complete growth medium and aspirate cells by gently pipetting. Add appropriate aliquots of the cell suspension to new culture vessels.
Split Time	2~3min
Subcultivation Ratio	1:3-1:4
Doubling Time	~24-45 hours
Medium Renewal	every 2 to 3 days
Cryopreservation	Freeze Medium: 55% Basal Medium+40% FBS+5% DMSO<br>Storage Temperature: Liquid Nitrogen Vapor Phase
Culture Conditions	Atmosphere: Air, 95%; CO2, 5%;<br>Temperature: 37℃
**3.Special Features of the Cell Line**	
Tumorigenic	Yes
Receptor Expression	
Antigen Expression	
Applications	This cell line is a suitable transfection host.
Cell Line Collections	ATCC; CRL-1932<br>BCRC; 60243

使用前请仔细阅读说明书。如果有任何问题，请通过以下方式联系我们：

全国免费电话：400-650-3656
销售电话：027-87287608
销售邮箱：sales@procell.com.cn
官方网站：www.procell.com.cn

武汉普诺赛生命科技有限公司

Procell Life Science&Technology Co,.Ltd.

786-0 [786-0]细胞出库质检单

一、产品信息

1. 细胞名称：786-0 [786-0]（人肾透明细胞腺癌细胞）
2. 细胞货号：CL-0010
3. 出库日期：2021-12-21

二、检测项目及结果

检测项目	检测结果	
生长特性（贴壁/悬浮）	贴壁	
细胞形态	上皮细胞样	
细胞密度	＞75%	
细胞纯度	——	
细胞总量	～1×10^6/Cells	
细胞活力	＞95%	
HIV-1	有☐	无☑
HBV	有☐	无☑
HCV	有☐	无☑
支原体	有☐	无☑
细菌	有☐	无☑
酵母	有☐	无☑
真菌	有☐	无☑

三、质检员及质检日期

质检员：王蕾　　质检日期：2021-12-18

质量合格 准予放行

武汉普诺赛生命科技有限公司 质检专用章

网站：www.procell.com.cn
电话：400-650-3656
邮箱：sales@procell.com.cn

附图 1：ACHN 细胞 STR 位点和 Amelogenin 位点的基因分型

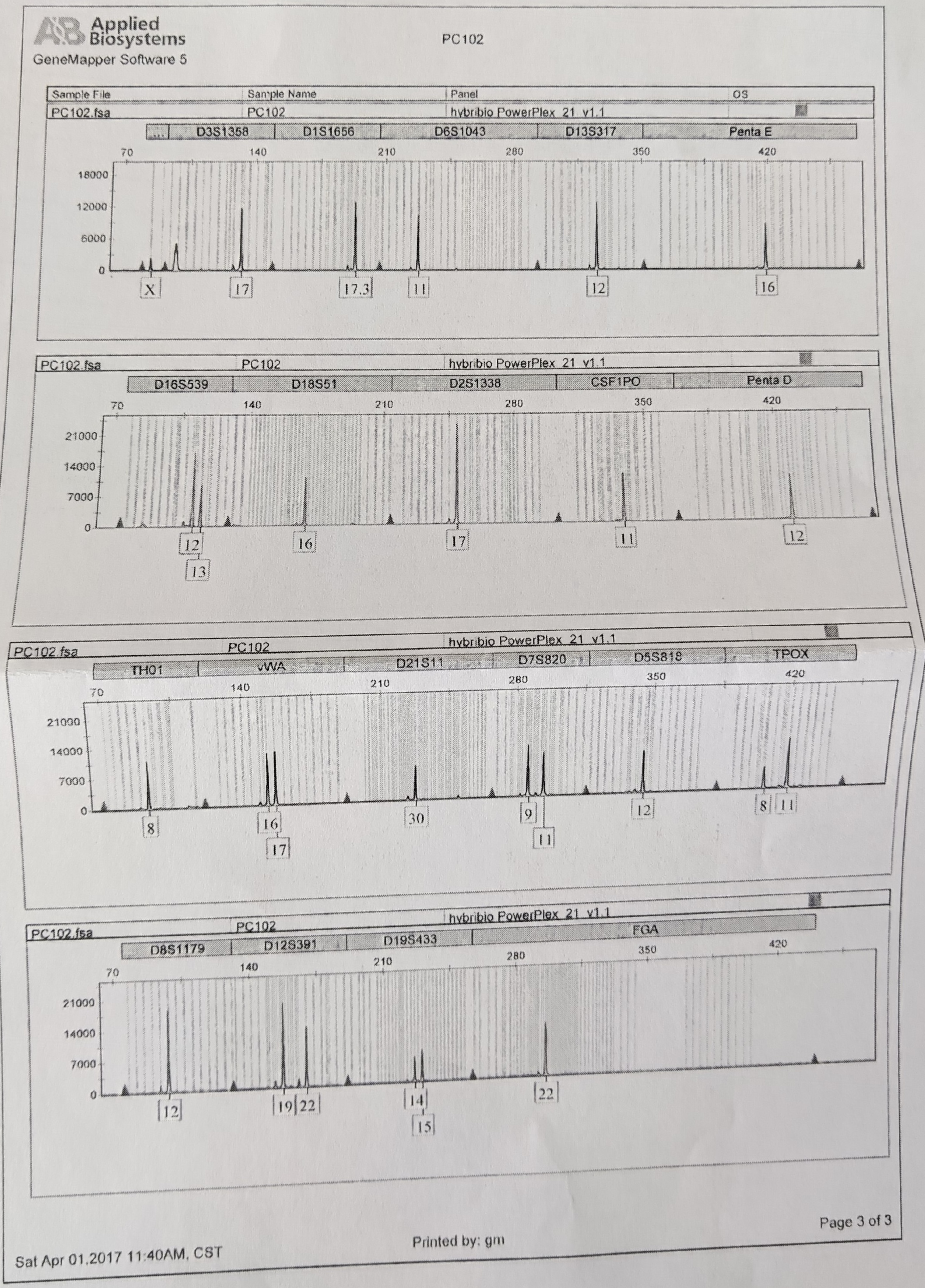

销售电话：400-650-3656
企业 QQ：4006503656
销售邮箱： sales@procell.com.cn
官方网站： www.procell.com.cn

附表 1：细胞株 ACHN 的 STR 位点和 Amelogenin 位点的基因分型结果

STR Loci	样品名称：PC102	数据库名称：ACHN
Amelogenin	X	X
CSF1PO	11	11
D2S1338	17	17
D3S1358	17	17
D5S818	12	12
D7S820	9,11	9,11
D8S1179	12	12
D13S317	12	12
D16S539	12,13	12,13
D18S51	16	16
D19S433	14,15	14,15
D21S11	30	30
FGA	22	22
PentaD	12	12
PentaE	16	16
TH01	8	8
TPOX	8,11	8,11
vWA	16,17	16,17
D1S1656	17.3	
D6S1043	11	
D12S391	19,22	
D2S441		
Cellosaurus 数据库匹配度 100.00%，匹配位点数 17（https://web.expasy.org/cellosaurus-str-search/）		

备注：

1. 根据国际细胞鉴定委员会(ICLAC)制定的细胞 STR 鉴定标准，细胞系的匹配度≥80%时，认为它们具有相关性，即衍生于共同的祖先细胞；匹配度在 55% 至 80% 之间，需要进一步验证相关性；小于 55%，表明两者不具有相关性。
2. 图谱有效峰为真实的 PCR 条带;小峰和非特异性条带在计算中忽略不计。
3. STR 数据比对结果默认 ExPASy，数据来源包括 ATCC，DSMZ，JCRB 等细胞库以及文献和资料记载，数据库入口 https://web.expasy.org/cellosaurus-str-search/。

ACHN 细胞 STR 鉴定报告

一、 材料处理和检验方法

取适量 **ACHN** 细胞(编号 PC102,1×10^6)使用 Chelex100 提取 DNA，采用 21 CELLID System 扩增 20 个 STR 位点和性别鉴定位点，使用 ABI3130x1 型遗传分析仪进行 PCR 产物检测，使用 GeneMapper IDX 软件（Applied Biosystems）对检测结果进行分析，并与 ATCC、DSMZ、JCRB、Cellosaurus 等数据库进行比对。

二、 检测结果

实验中阴性及阳性对照结果均正确。

ACHN 细胞株的 STR 位点和 Amelogenin 位点的基因分型结果见附表，分型图谱见附图。

三、 分析说明

ACHN 细胞株基因组 DNA 扩增后图谱清晰，分型结果良好。

四、 检验结论

1. **ACHN** 细胞株 DNA 进行细胞 STR 分型结果显示，细胞株中未发现人类细胞交叉污染。
2. 该细胞株 DNA 分型在细胞库中找到与其细胞分型 100.00%相匹配的细胞株，细胞株名称为 **ACHN**。

www.procell.com.cn

销售电话：400-650-3656
企业 QQ：4006503656
销售邮箱：sales@procell.com.cn
官方网站：www.procell.com.cn

CL-0021

ACHN（人肾细胞腺癌细胞）

**1.Origin and General Characteristics**	
**Cell Name**	ACHN
**Synonyms**	
**Organism**	Homo sapiens, Human
**Age**	22 years
**Tissue**	Kidney, derived from metastatic site: pleural effusion
**Morphology**	Epithelial
**Growth Properties**	Adherent
**Descriptions**	Growth is inhibited by human interferon [Hogan, T. F].
**Biosafety Level**	1
**2.Culture Conditions and Handling**	
**Complete Growth Medium**	MEM (PM150410) + 10% FBS (164210-500) + 1% P/S (PB180120)
**Subculturing**	Remove and discard culture medium. Briefly rinse the cell layer with DPBS solution to remove all traces of serum that contains trypsin inhibitor.<br>Add 1.0 to 2.0 mL of Trypsin-EDTA solution to flask and observe cells under an inverted microscope until cell layer is dispersed (usually within 2~3min). Cells that are difficult to detach may be placed at 37℃ to facilitate dispersal.<br>Add 4.0 to 6.0 mL of complete growth medium and aspirate cells by gently pipetting. Add appropriate aliquots of the cell suspension to new culture vessels.
**Split Time**	2~3min
**Subcultivation Ratio**	1:2-1:4
**Doubling Time**	~28-36 hours
**Medium Renewal**	every 2 to 3 days
**Cryopreservation**	Freeze Medium: 55% Basal Medium+40% FBS+5% DMSO<br>Storage Temperature: Liquid Nitrogen Vapor Phase
**Culture Conditions**	Atmosphere: Air, 95%; CO2, 5%;<br>Temperature: 37℃
**3.Special Features of the Cell Line**	
**Tumorigenic**	Yes
**Receptor Expression**	
**Antigen Expression**	
**Applications**	ACHN may be of use for antiproliferative studies using human interferons or interferon inducers.
**Cell Line Collections**	ATCC; CRL-1611<br>ECACC; 88100508

使用前请仔细阅读说明书。如果有任何问题，请通过以下方式联系我们：

全国免费电话：400-650-3656
销售电话：027-87287608
销售邮箱：sales@procell.com.cn
官方网站：www.procell.com.cn

武汉普诺赛生命科技有限公司

Procell Life Science&Technology Co,.Ltd.

ACHN 细胞出库质检单

一、产品信息

1. 细胞名称：ACHN（人肾细胞腺癌细胞）
2. 细胞货号：CL-0021
3. 出库日期：2021-12-28

二、检测项目及结果

检测项目	检测结果	
生长特性（贴壁/悬浮）	贴壁	
细胞形态	上皮细胞样	
细胞密度	＞75%	
细胞纯度	——	
细胞总量	～1×10^6/Cells	
细胞活力	＞95%	
HIV-1	有☐	无☑
HBV	有☐	无☑
HCV	有☐	无☑
支原体	有☐	无☑
细菌	有☐	无☑
酵母	有☐	无☑
真菌	有☐	无☑

三、质检员及质检日期

质检员：质量合格 准予放行 王悍　　质检日期：2021-12-14

武汉普诺赛生命科技有限公司 质检专用章

网站：www.procell.com.cn
电话：400-650-3656
